# Establishment an echovirus 6 infection model based on hFcRn transgenic mice

**DOI:** 10.3389/fmed.2025.1657471

**Published:** 2025-10-20

**Authors:** Fei Li, Yiliang Fu, Luci Huang, Hanwen Zhang, Juan Huang, Yi Wang, Zhengde Xie, Xiangpeng Chen

**Affiliations:** Beijing Key Laboratory of Core Technologies for the Prevention and Treatment of Emerging Infectious Diseases in Children, Laboratory of Infection and Virology, Ministry of Education, National Clinical Research Center for Respiratory Diseases, Research Unit of Critical Infection in Children, 2019RU016, Chinese Academy of Medical Sciences, National Center for Children’s Health, Beijing Children’s Hospital, Beijing Pediatric Research Institute, Capital Medical University, Beijing, China

**Keywords:** echovirus 6, hFcRn, transgenic mouse, pathogenesis, neuropathology

## Abstract

Echovirus 6 (E6) infection, a member of enterovirus, can cause severe neurological complications, particularly viral meningitis and encephalitis in children. However, the shortage of effective animal models has substantially impeded research on its pathogenesis and the advancement of therapeutic strategies. This study established and characterized a novel E6 infection model by employing transgenic (Tg) mice carrying the human neonatal Fc receptor (hFcRn). Following intracranial injection via the foramen magnum with E6, hFcRn^Tg^ mice exhibited significantly lower survival rates, impaired weight gain, and more severe clinical manifestations compared to wild-type control. Elevated levels of virus were detected in the brain, spinal cord, and muscle tissues of hFcRn^Tg^ mice, accompanied by substantial pathological changes, including neuronal damage, glial cell proliferation, and inflammatory infiltration. Immunofluorescence analyses confirmed active viral replication in the thalamus, meninges, and hippocampus. Extensive cytokine analysis showed increased concentrations of pro-inflammatory mediators, including MCP-1, IFN-γ, and TNF-α. Transcriptomic and proteomic analyses revealed enhanced immune pathways and suppressed metabolic processes, with key proteins MyD88, Cxcl10, and Irf3 central to the host response. Notably, our findings suggest that E6 infection may engage ZBP1-centered PANoptosis, although the underlying mechanisms require further validation. This model provides a valuable tool for investigating E6 pathogenesis and evaluating potential therapeutic strategies.

## 1 Introduction

Echovirus 6 (E6), classified within the *Enterovirus betacoxsackie* species of the genus Enterovirus in the *Picornaviridae* family, is a single-stranded, positive-sense RNA virus. Its genome spans approximately 7.4 kb and encodes 11 viral proteins: VP1, VP2, VP3, VP4, 2A, 2B, 2C, 3A, 3B, 3C, and 3D (1, 2). Based on the VP1 sequence characteristics, E6 can be classified into six subgenotypes (A-F), with subgenotypes E and F being the primary types associated with acute flaccid paralysis (AFP) and viral meningitis (VM) in children (3). From 1988 to 2018, the predominant circulating E6 subgenotypes in China were C, E, and F, with subgenotype F becoming the most prevalent since 2007 (3).

E6 infection can result in a range of clinical manifestations in children, with mild cases presenting as fever and respiratory symptoms, while severe cases may present with central nervous system (CNS) symptoms, potentially leading to serious complications or even death (4–8). Between April and July 2005, an outbreak of aseptic meningitis linked to E6 was reported in Jinzhai County, Anhui Province, impacting 97 children between the ages of 3 and 15. Thirty (30.93%) E6 strains were isolated and exhibited a genetic homogeneity of 99.7 to 100% and were classified within the C4 subcluster. This is also the first reported outbreak of E6-associated meningitis in China (9). E6 has been reported as the predominant pathogen among 75 pediatric enterovirus encephalitis cases in Hunan province, China, and between 2017 and 2019, accounting for 52.0% (39/75) of infections (10). An outbreak of neurologically symptomatic E6 infections was documented in the Netherlands during the summer of 2016, with 15 out of 31 cases exhibiting neurological manifestations, predominantly clustered in a single province (6). An atypical transmission pattern of E6 meningitis was reported during the 2021–2022 COVID-19 pandemic in Israel, characterized by a 66% decline in cases during the Omicron wave, followed by a subsequent 78% surge, with E6 accounting for 29% of enterovirus cases and predominantly affecting patients around 25 years of age (7). Environmental surveillance by Susana Monge demonstrated the presence of E6 strains in wastewater and drinking water systems from 2007 to 2016. Genetic analysis uncovered that these environmental strains shared notable genetic similarity with clinical isolates implicated in outbreaks of VM and viral encephalitis (VE) among local children (11).

The pathogenic mechanisms of E6 infection leading to VM and VE remains to be elucidated, and there are presently no approved vaccines or therapies for prevention or treatment. Stable animal models are critical for studying pathogenic mechanisms, vaccine development, and drug discovery; however, no animal model for E6 infection is available to date. Humans are the only natural host of echoviruses. The human neonatal Fc receptor (hFcRn) was reported to be a key receptor in the recognition and mediation of echovirus infections, including E6 which provides the possibility for the establishment of transgenic animal model (12, 13). This study aims to construct a stable and effective E6 infection model using hFcRn transgenic mice (Tg). 7-day-old C57BL/6 hFcRn^Tg^ mice were utilized and simulated disease progression through intracranial injection of the E6. Clinical symptom scores and survival rates were recorded and analyzed in the infected mice. Furthermore, viral replication, as well as pathological and immunohistochemical features in the brain, spinal cord, and muscle tissues, were examined to confirm successful disease model establishment and to identify key biological markers.

## 2 Materials and methods

### 2.1 Animals, cells and viruses

hFcRn^Tg^ mice, C57BL/6 J strain were specific pathogen free (SPF) and were kindly provided by Academician Gao Fu’s research group. All hFcRn^Tg^ mice were kept in separate ventilated enclosures and supplied with adequate food and water.

Human rhabdomyosarcoma (RD) (CL-0193, Pricella, Wuhan, China) cells were cultured in Dulbecco’s modified Eagle medium (DMEM) (G4511, Servicebio, Wuhan, China) containing 10% fetal bovine serum (FBS) (10,099,141, Gibco, New York, USA) and 1% penicillin–streptomycin (PS) (15,140,122, Gibco, New York, USA) at 37 °C with 5% CO2. RD cells were used to propagate this virus. Viruses in this study included E6-SJZ366 (GenBank: MH830353.1), which was from the cerebrospinal fluid (CSF) sample of a patient with encephalitis.

### 2.2 Animal infection experiments

Seven-day-old hFcRn^Tg^ mice were infected with E6 strain SJZ366 at 4.0 × 10^7^ PFU through intracerebral (IC) routes; control mice were inoculated with maintenance medium (MM) and reared separately from the infected mice (*n* = 9 per group). The weights, survival rates, and clinical scores of the mice were recorded daily. The grade of clinical disease was scored as follows: 0, no disease; 1, significant weight loss; 2, inability to right itself when placed on back; 3, hind limb weakness; 4, left/right hind limb paralysis; 5, mortality.

### 2.3 Determination of virus titers in mouse tissues

Mouse tissues were collected from 7-day-old hFcRn^Tg^ mice via IC injection of SJZ366 at 1 to 7 days post-infection (dpi). All samples were suspended in phosphate-buffered saline (PBS) and homogenized using an automated tissue grinder (SWE-C6, Servicebio, Wuhan, China). After three freeze–thaw cycles, the mixtures were centrifuged to obtain the supernatant. At each time point, the collected supernatant was subjected to 10-fold serial dilutions across 12 gradients and inoculated into RD cells seeded in 96-well plates. Following 7 days of incubation and observation, viral titers were calculated using the TCID_50_ assay.

### 2.4 Sample collection and preprocessing for cytokine analysis

Brain tissues were collected and weighed. For tissue homogenization, 500 μL of ProcartaPlex Cell Lysis Buffer (containing protease inhibitors) (EPX-99999-000, Thermo Fisher Scientific, Waltham, MA, USA) was added per 100 mg of tissue. Tissues were homogenized using a mechanical homogenizer with 5 mm stainless steel beads at 25 Hz for 0.5–3 min. After centrifugation at 16,000 × g for 10 min at 4 °C, the supernatant was collected. Total protein was quantified and normalized to 10 mg/mL using 1 × PBS before storage at −80 °C.

### 2.5 Multiplex cytokine analysis

Cytokine concentrations were determined with the ProcartaPlex Mouse Cytokine & Chemokine 36-Plex Panel 1a (EPX360-26092-901, Thermo Fisher Scientific, Waltham, MA, USA) array. Thirty-six cytokines were simultaneously analyzed, including interferon-gamma (IFN-γ), interleukin (IL) series, tumor necrosis factor-alpha (TNF-α), and chemokines. Briefly, magnetic beads were prepared and washed with assay buffer. Standards were reconstituted and serially diluted (4-fold, with 8 concentrations). Portions of brain tissue samples were collected and analyzed for viral presence using the TCID_50_ method, ensuring that the experimental group was indeed under viral infection conditions. The remaining brain tissue samples were homogenized in ProcartaPlex Cell Lysis Buffer (EPX-99999-000), and the supernatant was subsequently collected for further analysis. Blood was collected in endotoxin-free tubes, clotted 20–30 min at room temperature, then centrifuged at 1,000 × g for 10 min at 4 °C. Serum was aliquoted and stored at −80 °C until analysis. Samples (25 μL for diluted tissue homogenates) and standards were incubated with the beads in a 96-well plate at room temperature for 30 min with shaking (500 rpm), followed by overnight incubation at 4 °C. After washing, detection antibodies were added and incubated for 30 min at room temperature. After washing, the plate was incubated with Streptavidin-Phycoerythrin (SA-PE) for 30 min. Subsequently, beads were washed again, resuspended in Reading Buffer, and analyzed using the Luminex 200 system. Cytokine concentrations were calculated using five-parameter nonlinear regression analysis of the standard curves.

### 2.6 Histopathological and immunofluorescence assays

At 2 dpi following SJZ366 IC injection in 7-day-old hFcRn^Tg^ mice, brains from both groups (*n* = 3 per group) were harvested, fixed in formalin for 24 h, dehydrated, and paraffin-embedded. Five-micrometer sections were cut, deparaffinized with xylene, and stained with H&E. Imaging and analysis were performed using the PANNORAMIC DESK/MIDI/250/1000 scanner and CaseViewer 2.4 software (3DHISTECH, Budapest, Hungary). Tissues designated for immunofluorescence were fixed in 10% paraformaldehyde for 24 h, embedded in OCT, and cryosectioned. To evaluate E6 double-stranded RNA (dsRNA) distribution, sections were incubated overnight at 4 °C with mouse polyclonal anti-dsRNA J2(1:500, 10,010,500, SCICONS, Szirák, Hungary) and mouse monoclonal anti-GAPDH (1:100, GB15002, Servicebio, Wuhan, China) antibodies.

### 2.7 Library preparation for transcriptome sequencing

E6 (4.0 × 10^7^ PFU) was administered by intracerebral injection into 7-day-old hFcRn^Tg^ and control mice (*n* = 3 per group). Brain tissues were harvested for total RNA extraction with TRIzol reagent (15,596,026, Invitrogen, CA, USA). The RNA integrity was assessed by agarose gel electrophoresis, and RNA concentration was accurately quantified using a NanoDrop spectrophotometer; mRNA was isolated from total RNA with oligo-dT-onjugated magnetic beads and subsequently fragmented; without pre-fixing the insert size, first- and second-strand cDNA were synthesized using reverse transcriptase, after which the reverse-transcription products underwent end repair and 3′ adenylation to generate double-stranded libraries; sequencing adapters were ligated to the fragments, and the ligation products were purified to remove incompletely ligated molecules and adapter dimers; PCR amplification was then performed using primers complementary to the adapter sequences, and the amplified libraries were purified with magnetic beads to yield the final sequencing libraries; following library construction, library concentration was measured with a Qubit fluorometer and fragment-size distribution was evaluated using an Agilent fragment analyzer to ensure library quality; libraries that passed quality control were sequenced on the Illumina NovaSeq 6,000 platform using a PE150 strategy, wherein 150 bp are read from each end of the fragments. Each sample is assigned its own unique set of indexes (non-overlapping across samples), and demultiplexing is performed after sequencing based on the index information. (NCBI BioProject ID: PRJNA1331447, to be released in 2026).

### 2.8 RNA-Seq data bioinformatics analysis

After quality control with fastp (v0.20.1), the cleaned reads were aligned to the mouse reference genome GRCm38.p5 (mm10) using STAR (v2.7.8a), with the genome index built from Gencode vM13 annotations. Gene-level quantification was performed with RSEM (v1.3.3) to obtain raw counts and TPMs. Differential expression analysis was conducted with edgeR (v3.28.1) using raw counts with TMM normalization and statistical testing, applying a significance threshold of *p* < 0.05 and |log2 fold change| ≥ 1.5 to identify differentially expressed genes (DEGs). Functional enrichment was performed with clusterProfiler (v3.14.3) using annotations from org.Mm.eg.db (v3.10.0) and KEGG Release 102.

### 2.9 Sample preparation for proteome sequencing

Seven-day-old hFcRn^Tg^ and control mice received IC injections of E6 (4.0 × 10^7^ PFU). Brain tissue was lysed on ice in RIPA buffer supplemented with protease and phosphatase inhibitors, followed by centrifugation to collect the supernatant. Portions of brain tissue samples were collected and analyzed for viral presence using the TCID_50_ method, ensuring that the experimental group was indeed under viral infection conditions. Protein concentration was determined by the BCA assay, and equal amounts of protein were processed by reduction with DTT/TCEP and alkylation with IAA/CAA, then digested with trypsin at 37 °C overnight at an enzyme-to-substrate ratio of approximately 1:50–1:100. The digests were acidified, desalted using C18 SPE, and reconstituted in 0.1% formic acid. A label-free quantification strategy was employed without isotopic tagging, and the primary workflow used single-shot analysis without prefractionation. Peptides were separated by nano-flow reversed-phase LC using a water/acetonitrile gradient containing 0.1% formic acid and analyzed on a Q Exactive HF-X mass spectrometer in data-dependent acquisition (DDA) mode. Raw MS data were processed with MaxQuant (version 1.6.6) using the SwissProt database for protein identification. The protein database was obtained from the UniProt database.

### 2.10 Proteomics data bioinformatics analysis

Sample-specific protein database was constructed, and raw mass spectrometry data were searched against it using MaxQuant. Based on UniProt-derived search results, quality control was performed at the peptide and protein levels. Identified proteins were annotated using standard resources, including GO, KEGG, protein domains, COG/KOG, and STRING. Protein quantification, differential protein identification, and functional classification were then conducted. Label-free quantification (LFQ) was applied, and proteins with *p* < 0.05 and a fold change of ≥1.5 were considered differentially expressed. Enrichment of biological functions and pathways among differentially expressed proteins was assessed using Fisher’s exact test. Finally, protein–protein interaction (PPI) network analysis was performed to identify key regulatory proteins under the experimental conditions.

### 2.11 Quantification and statistical analysis

Statistical tests and generation of graphs was done in GraphPad Prism 8.0. Mouse survival rates were compared using the log-rank (Mantel-Cox) test, while clinical scores were evaluated by the Wilcoxon test. Significance levels are denoted as follows: ^*^p < 0.05; ^**^p < 0.01; ^***^p < 0.001; ^****^p < 0.0001; and “ns” for non-significant results. Final figures were edited and assembled in Adobe Illustrator.

## 3 Results

### 3.1 Establishment of an E6 hFcRn^Tg^ mouse model of encephalitis infection

**Figure 1:**
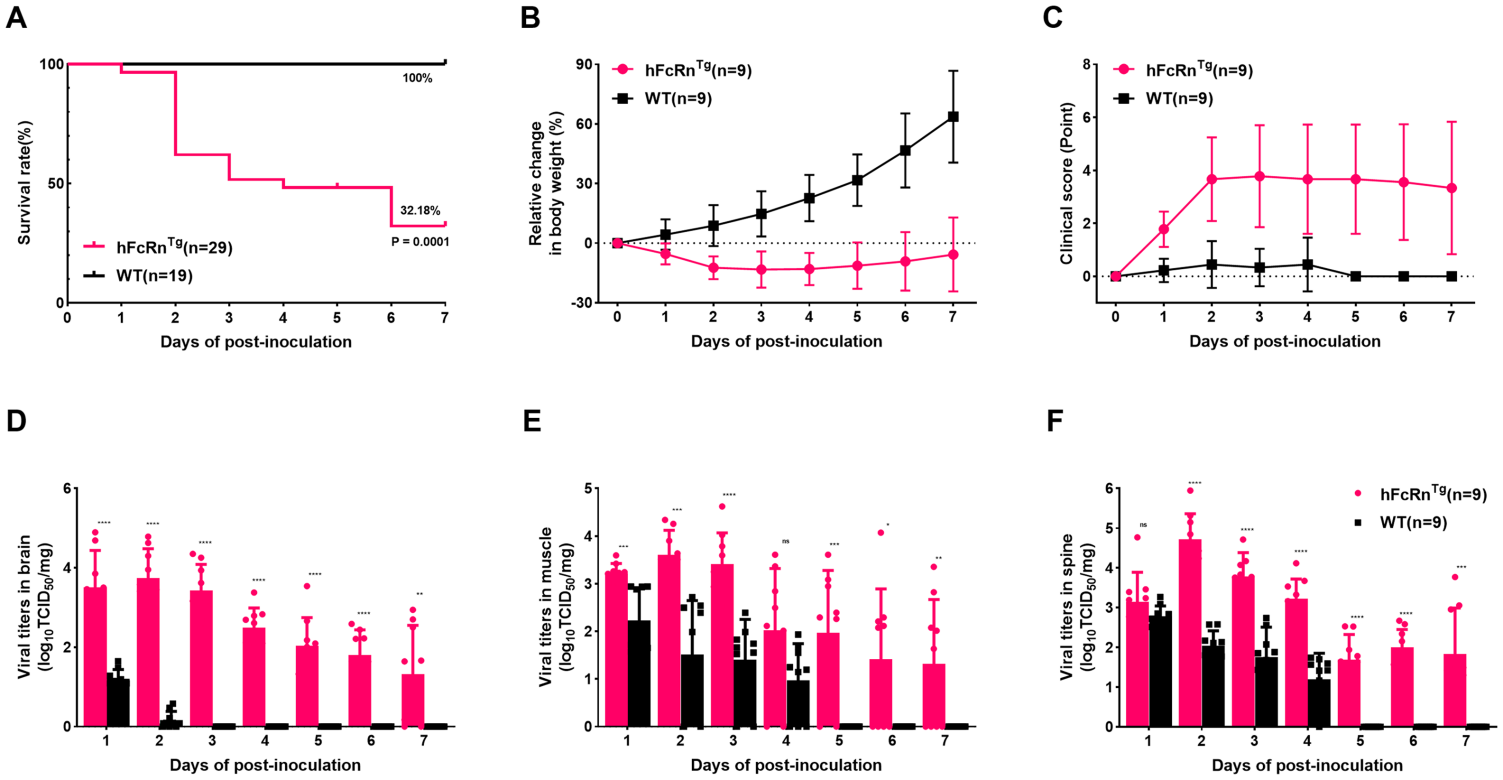
Establishment of the E6 infection mouse encephalitis model. **(A)** Survival rates of hFcRn^Tg^ and WT mice infected with E6 (4.0 × 10^7^ PFU) at 7 days old via IC route. **(B)** Body weight changes in mice infected through IC route over 7 days post-infection. **(C)** Clinical scores of mice over 7 dpi. Temporal dynamics of viral titers in different tissues over 7 dpi by TCID_50_ methods: **(D)** Brain, **(E)** Muscle, and **(F)** Spine. ^*^p < 0.05; ^**^p < 0.01; ^***^p < 0.001; ^****^p < 0.0001; and “ns” for non-significant results.

The survival analysis revealed that hFcRn^Tg^ mice had a significantly lower survival rate compared to wild-type (WT) mice over the 7 days observation period following infection (*p* = 0.0001, 32.18% vs. 100%) (Figure 1A). Additionally, the hFcRn^Tg^ group showed no significant increase in body weight, whereas WT mice exhibited a stable growth trajectory, with body weight increasing to approximately 60% by 7 dpi (Figure 1B). Clinical scoring further supported these findings, as hFcRn^Tg^ mice displayed elevated scores, peaking at around 4 points by 3 dpi, indicating more severe clinical manifestations (Figure 1C). In terms of viral load, hFcRn^Tg^ mice exhibited significantly higher viral titers in the brain on 1 to 3 dpi, suggesting enhanced viral proliferation in the CNS (Figure 1D). Similarly, viral titers in muscle and spinal cord samples showed that hFcRn^Tg^ mice had significantly higher viral loads compared to WT mice on 2 and 3 dpi (Figures 1E,F).

### 3.2 Pathological and immunofluorescence characteristics in E6-infected hFcRn^Tg^ mice

**Figure 2:**
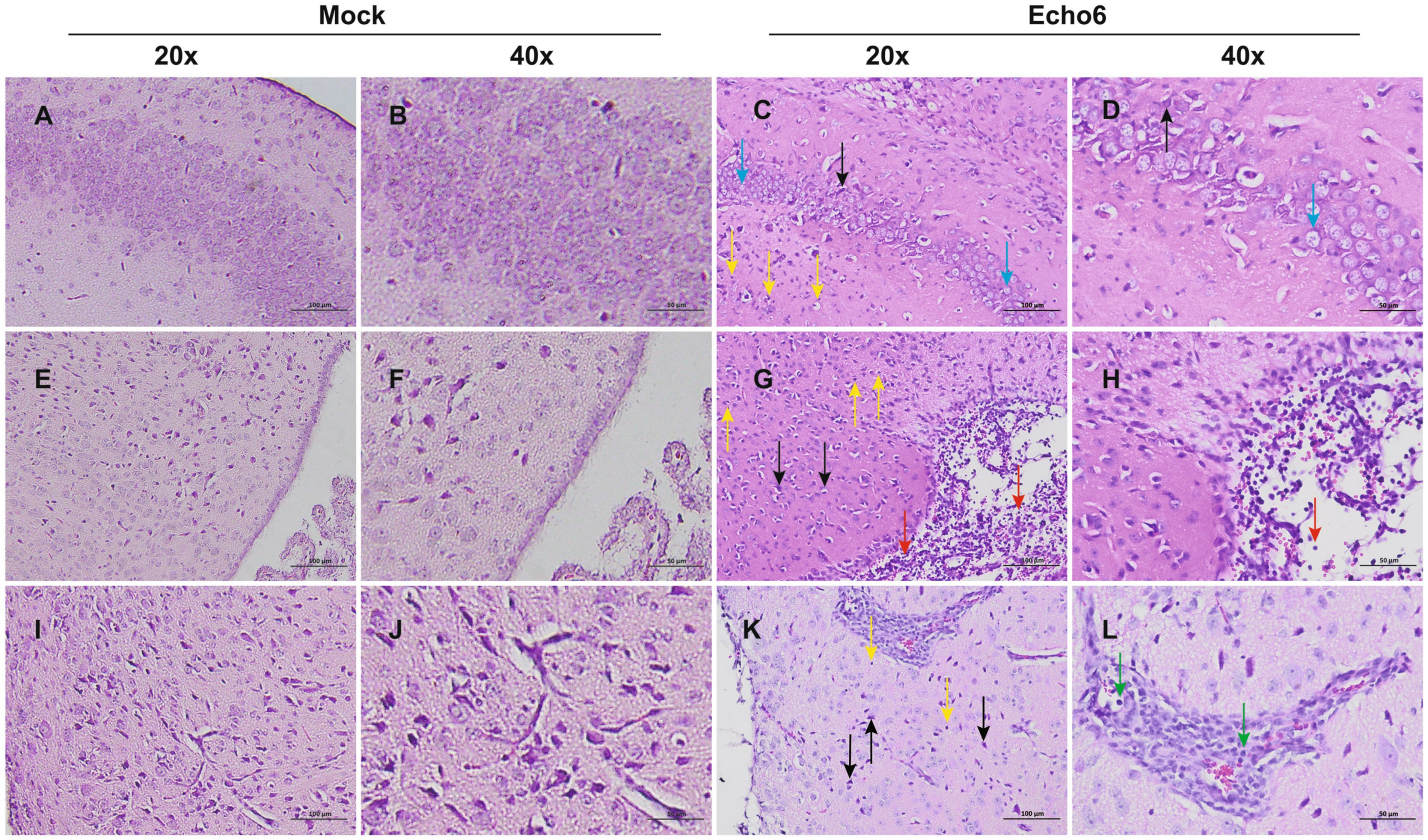
Histopathological changes of brain in mice. Representative H&E staining of brain tissue sections from mock (*n* = 3) and E6-infected (*n* = 3) mice at 2 dpi; Mock (**A,B,E,F,I,J**): uninfected control; Echo6 (**C,D,G,H,K,L**): E6-infected mice. The background color differences, due to minor variations in H&E staining batches, do not affect the histological interpretation. Scale bars: 20 × = 100 μm; 40 × = 50 μm.

**Figure 3:**
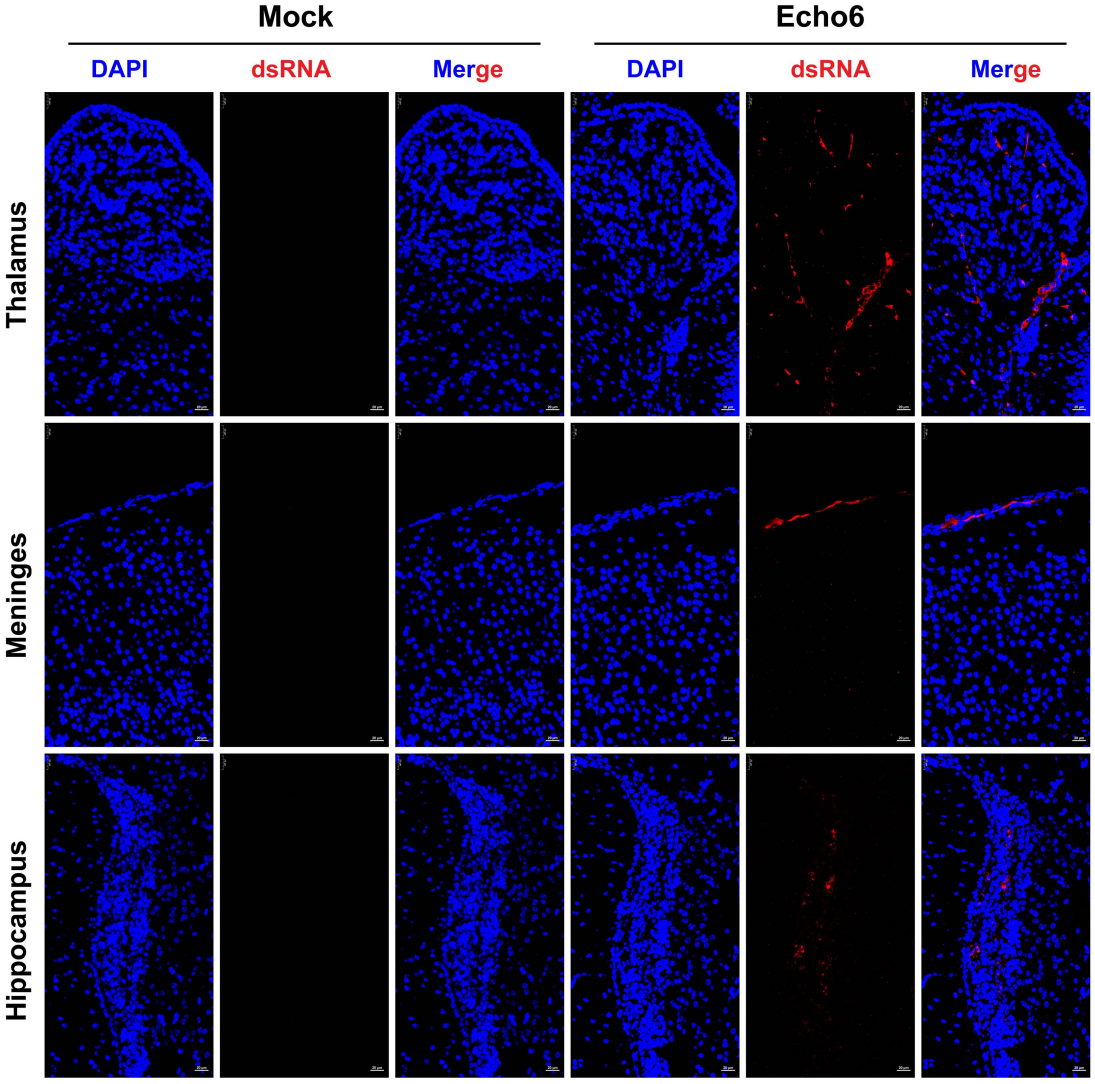
The proliferation of the E6 in mouse brain tissue. Mice were injected with E18 or cell culture maintenance medium. Brain tissue sections of suckling mice were fixed and immunostained with DAPI (blue, nuclear staining), dsRNA (red, viral double-stranded RNA). Mock: uninfected control; Echo6: E6-infected mice. Scale bar = 25 μm.

Histopathological evaluation of tissues revealed substantial architectural changes in E6-infected mice (Figures 2A–L), with some neurons exhibiting shrunken nuclei, deep staining, and unclear nuclear-cytoplasmic boundaries (black arrows). Evidence of slight glial cell proliferation (yellow arrows), enlarged pericellular spaces (blue arrows), and occasional perivascular cuffing forming vascular sleeves (green arrows) was also observed. Additionally, localized mild hemorrhaging in the meninges was noted, along with significant lymphocytic focal infiltration (red arrows) and signs of capillary congestion (orange arrows). Immunofluorescence analysis of hFcRn^Tg^ mice infected with E6 revealed significant viral replication compared to controls (Figure 3). In the thalamus, meninges, and hippocampus, mock samples showed no detectable dsRNA, whereas E6-infected tissues exhibited prominent dsRNA signals, indicating active viral presence.

### 3.3 Inflammatory response in infected mice brain

**Figure 4:**
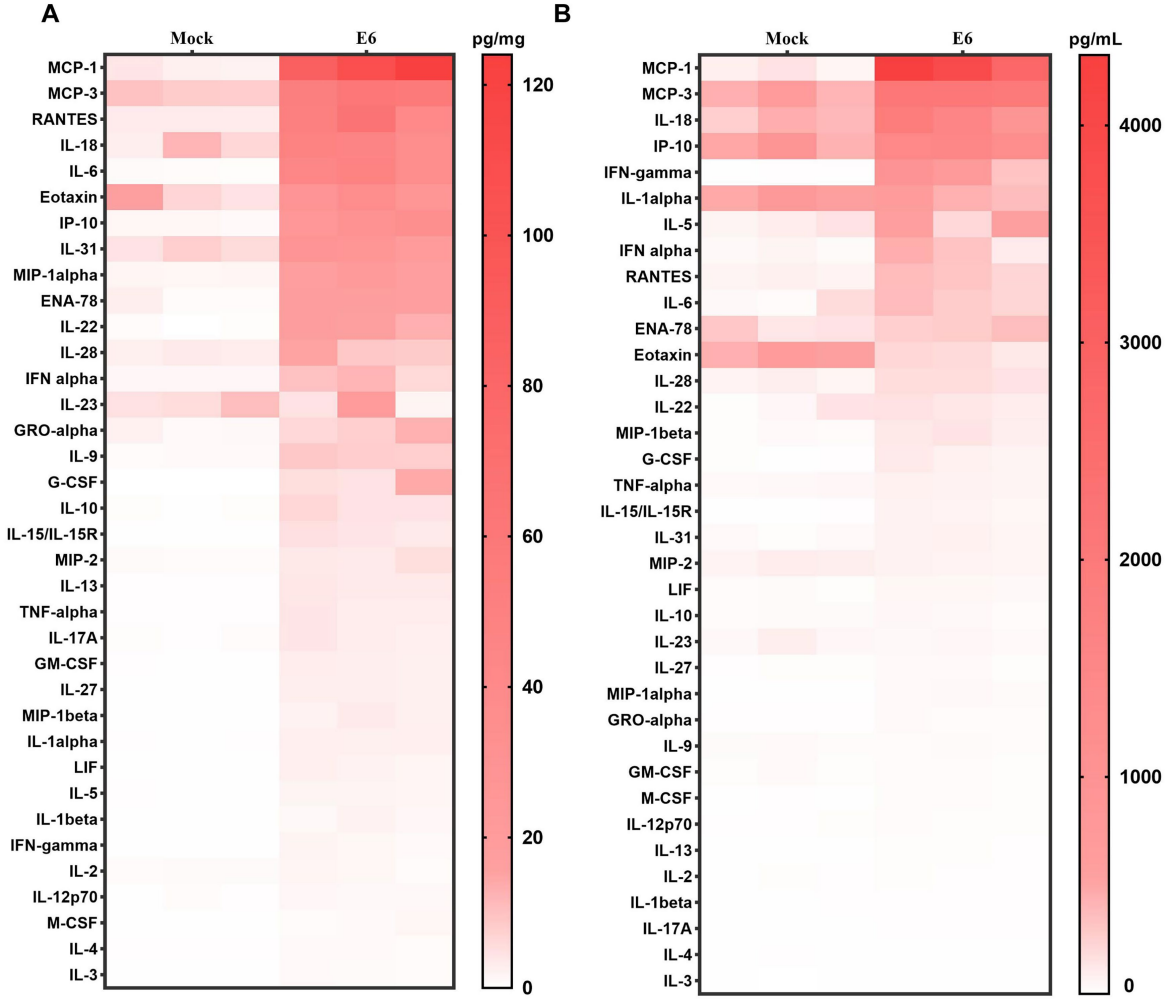
Inflammatory cytokine profiles in mice. Heat map showing cytokine/chemokine expression profiles in brain tissue (pg/mg) **(A)** and serum (pg/ml) **(B)** at 2 dpi, Color intensity indicates concentration.

Cytokine analysis of brain tissue in hFcRn^Tg^ mice infected with E6 showed significant changes in cytokine expression compared to mock-infected controls (culture medium) (Figure 4A). Notably, pro-inflammatory cytokines such as Interleukin-27 (IL-27), IFN-*γ*, and TNF-*α* were markedly elevated in E6-infected mice, suggesting a robust innate antiviral immune response. Additionally, chemokines such as monocyte chemoattractant protein-1 (MCP-1), monocyte chemoattractant protein-3 (MCP-3), and regulated on activation, normal T expressed and secreted (RANTES) showed significant increases, suggesting enhanced recruitment of immune cells to the site of infection. Other cytokines, including Interleukin-6 (IL-6), Interleukin-8 (IL-18), and Interleukin-9 (IL-9), also exhibited elevated levels, further supporting the presence of a heightened inflammatory environment. The serum cytokine profile also shows that E6 infection elicits a broadly upregulated systemic inflammatory response (Figure 4B). Among the elevated mediators, including MCP-1, MCP-3, IL-18, Interferon gamma–induced protein 10 (IP-10), and Interferon gamma (IFN-γ), MCP-1 and IFN-γ increased the most. Specifically, MCP-1 reached approximately 39-fold of the control in serum and approximately 38-fold in brain tissue, whereas IFN-γ reached approximately 124-fold and 15-fold of the control, respectively (Supplementary material 1).

### 3.4 Transcriptomic profiling reveals dynamic host response to E6 infection in the brain

**Figure 5:**
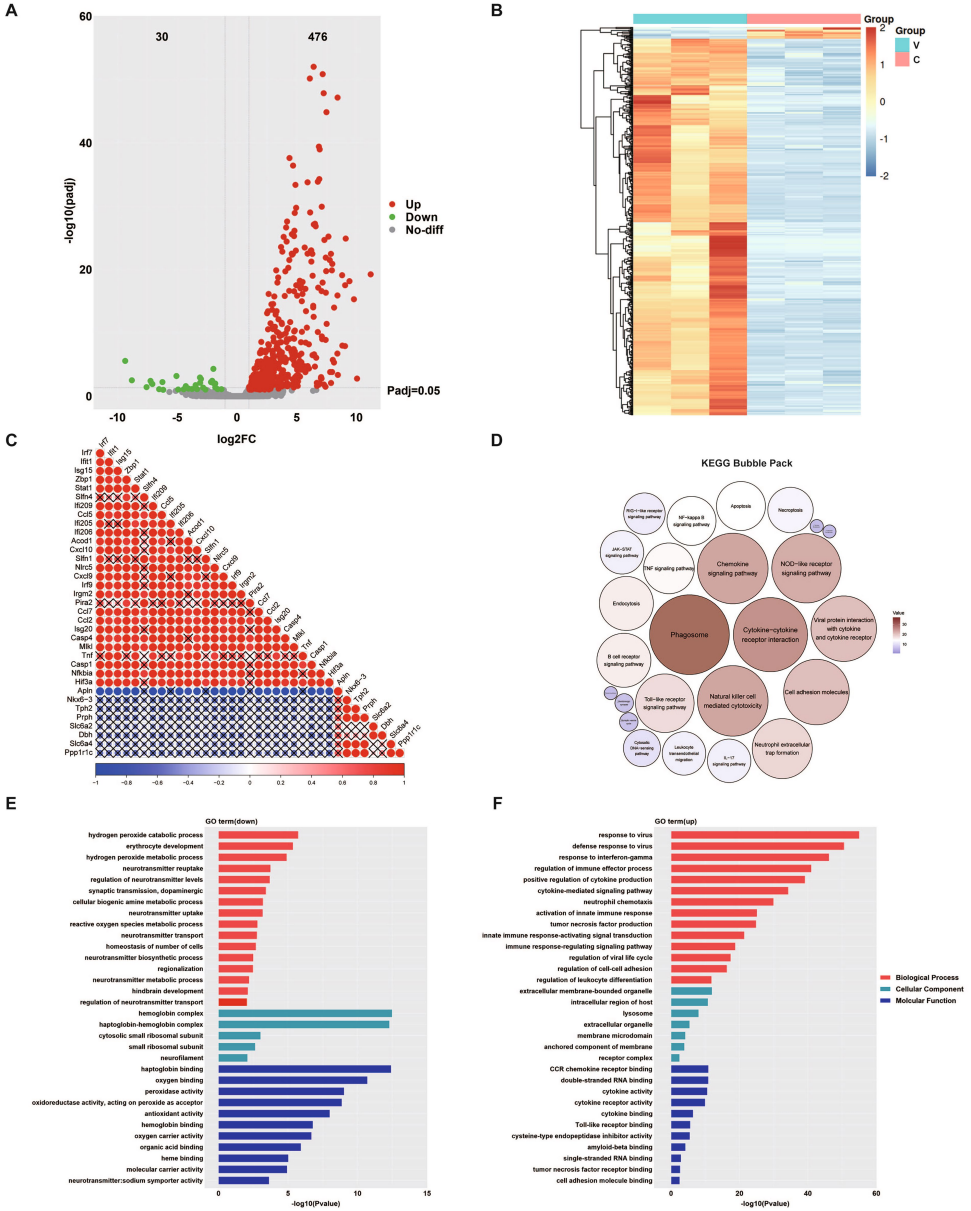
Transcriptomic analysis of E6-infected brain tissue. **(A)** Volcano plot displaying differentially expressed genes (DEGs). Red dots represent significantly upregulated genes, green dots indicate significantly downregulated genes, and grey dots represent genes with no significant change. **(B)** Hierarchical clustering heatmap showing differentially expressed proteins (DEPs) between two groups. Red indicates upregulation while blue indicates downregulation. C: uninfected control; V: E6-infected mice. **(C)** Correlation analysis of differentially expressed genes; dot size and color intensity indicate the strength of correlation, with red representing positive correlation and blue representing negative correlation. A “×” symbol indicates non-significant correlation (*p* > 0.05). KEGG pathway enrichment bubble pack for upregulated and downregulated pathways, in which the five smallest circles with downward arrows indicate the downregulated pathways **(D)**. GO enrichment analysis of downregulated **(E)** and upregulated pathways **(F)** showing the top enriched terms in three categories: biological process (red), molecular function (blue), and cellular component (green).

Transcriptomic analysis of brain tissues from hFcRn^Tg^ mice infected with E6 revealed significant alterations in gene expression profiles compared to mock (culture medium)-infected controls (Figures 5A,B). The E6 group yielded 2,338, 8,875, and 1,989 viral reads across the three samples, respectively; by contrast, all three control samples contained zero viral reads (Supplementary material 1). Correlation analysis of representative differentially expressed genes showed that interferon/inflammation-related genes (e.g., interferon regulatory factor 7 (Irf7), interferon-induced protein with tetratricopeptide repeats 1 (Ifit1), interferon stimulated gene 15 (Isg15), C-X-C motif chemokine ligand 10 (Cxcl10), C-C motif chemokine ligand 5 (Ccl5)) were coordinately upregulated following viral infection and, together with zinc-finger antiviral protein 1 (Zbp1), NLR family CARD domain containing 5 (Nlrc5), and caspase 4 (Casp4), formed a highly coherent, positively correlated module. In contrast, genes involved in neurotransmission and neuronal function (e.g., tryptophan hydroxylase 2 (Tph2), peripherin (Prph), solute carrier family 6 member 2 (Slc6a2), dopamine beta-hydroxylase (Dbh)) constituted a separate cluster that was broadly and significantly negatively correlated with the inflammatory module (blue) (Figure 5C). Additionally, KEGG analysis following viral infection revealed prominent activation of immune pathways centered on phagosome, cytokine-cytokine receptor interaction, and chemokine signaling pathway (Figure 5D). Consistent with this, upregulated GO terms highlighted antiviral or interferon responses, cytokine-mediated signaling, and neutrophil chemotaxis, whereas downregulated categories encompassed neurotransmitter synthesis, transport, and reuptake, as well as dopaminergic synaptic transmission and biogenic amine metabolism (Figures 5E,F). These patterns indicate an inverse association between activation of immune pathways and suppression of neuron or monoamine-related genes.

### 3.5 Proteomic analysis reveals multi-dimensional molecular changes in the brain

**Figure 6:**
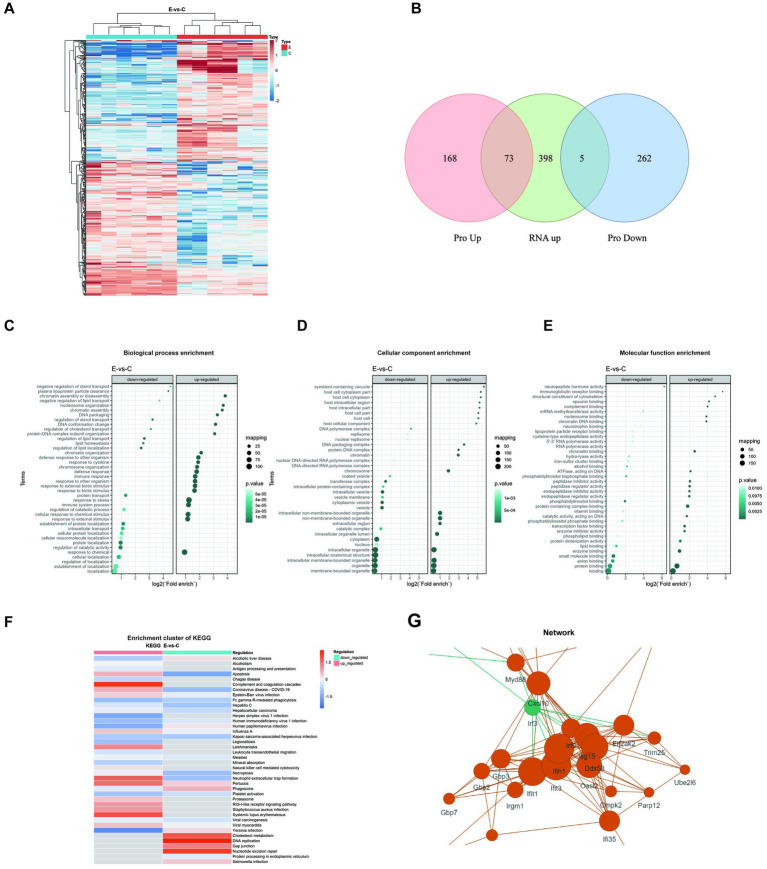
Proteomic analysis in E6-infected brain tissue. **(A)** Hierarchical clustering heatmap showing differentially expressed proteins (DEPs) between two groups. Red indicates upregulation while blue indicates downregulation. **(B)** Venn diagram showing overlaps between differentially expressed proteins (upregulated or downregulated) and upregulated genes. No panel for downregulated genes is included because no intersection was observed. Enrichment analysis of DEPs presented as bubble plots: **(C)** Biological process enrichment, **(D)** Cellular component enrichment, and **(E)** Molecular function enrichment. **(F)** KEGG cluster enrichment analysis comparing virus-infected versus control samples (E-vs-C), with color intensity indicating regulation levels. **(G)** Protein–protein interaction network of DEPs. All analyses were performed using data from three biological replicates per group, with statistical significance threshold set at *p* < 0.05.

Notably, protein expression profiles differed significantly between groups (Figure 6A). By integrating transcriptomic and proteomic data, we identified 73 upregulated proteins that were consistent with the transcriptomic findings, including signal transducer and activator of transcription 1/2 (Stat1/2), Isg15, Ifit1/3, dead-box helicase 58 (Ddx58), and interferon induced with helicase c domain 1 (Ifih1) et al. These proteins were enriched in interferon-mediated antiviral and innate immune pathways, covering pathogen recognition, antigen processing, and related inflammatory responses, thereby indicating an immune-activation state centered on interferon signaling (Figure 6B). Biological process enrichment analysis indicated downregulated processes such as lipid transport and upregulated processes related to defense responses (Figure 6C). Cellular component enrichment highlighted downregulated organelles and upregulated components linked to immune signaling (Figure 6D). Molecular function enrichment revealed downregulated catalytic and binding activities, while upregulated functions were associated with immune responses (Figure 6F). The enrichment cluster of KEGG pathways illustrated the interconnected pathways affected by E6 infection (Figure 6F), while network analysis identified key proteins, including myeloid differentiation primary response 88 (Myd88), Cxcl10, and interferon regulatory factor 3 (Irf3), as central to the immune response, underscoring their interactions (Figure 6G).

## 4 Discussion

E6 infection can lead to severe clinical manifestations, including acute flaccid paralysis and viral meningitis, particularly in children, underscoring the need for a reliable animal model to understand the pathogenic mechanisms. Numerous studies have developed murine models for CV-A16, EV-A71, and CV-A6 to investigate candidate vaccines and pathogenesis using ICR (Institute of Cancer Research) mice, and E30 by a wild 1-day-old mouse model (14–17). Our research group previously identified FcRn as the key functional receptor for enterovirus B, which facilitates viral uncoating and genome release upon cell entry (12). As humans serve as the exclusive natural hosts for EV, the significance of this receptor is heightened. However, there are species differences between human and mouse FcRn. This discovery has significantly advanced the development of transgenic mouse models for studying echovirus infections. For instance, Wells et al. utilized the hFcRn^Tg32-^IFNAR^−/−^ mouse model to demonstrate that the hFcRn and IFN signaling are critical factors in E11 infection (12). The IFN-I axis plays a key role in the protection against EV infection (18, 19). Wessely et al. (20) and Koestner et al. (21) showed that IFNAR^−/−^ mice are highly susceptible to coxsackievirus B3 (CV-B3). Moreover, Zhang et al. (22) established an E11-associated brain damage model via IC inoculation in 2-day-old IFNAR^−/−^ mice using a dose of 10^7^ TCID_50_ E11. However, immunodeficient mice, with their incomplete immune systems, fail to accurately represent the interactions between the virus and the host immune response, limiting their ability to mimic the pathological processes of natural human infections. This limitation introduces bias in evaluating the viral pathogenesis mechanisms and the efficacy of therapeutic interventions and vaccines.

The establishment of the E6 hFcRn^Tg^ mouse model underscores the critical role of the hFcRn receptor in the pathogenesis of E6 infection. This study found that mice in the E6 group exhibited increased mortality and pronounced weight loss at 2 dpi, while viral titers in the brain, muscle, and spinal cord peaked, indicating an acute phase of infection on 2 dpi followed by gradual recovery. The significantly lower survival rates and more severe clinical manifestations observed in hFcRn^Tg^ mice suggest that this receptor facilitates enhanced viral replication and neuroinvasion. This finding is consistent with previous studies demonstrating that Fc receptors play a significant role in modulating host susceptibility to viral infections (12, 13, 23, 24). The elevated viral loads in the CNS of hFcRn^Tg^ mice further support the hypothesis that hFcRn enhances viral entry and replication, leading to more severe disease outcomes. The pronounced differences in viral load and clinical outcomes between hFcRn^Tg^ and WT mice highlight the potential of this model for further investigations into the mechanisms of E6 neuroinvasion and the evaluation of antibody, drug, and vaccine efficacy for targeted therapeutic interventions.

The pathological and immunofluorescence findings in E6-infected hFcRn^Tg^ mice provide critical insights into the mechanisms of viral encephalitis. The observed architectural changes in brain parenchyma, including neuronal shrunken nuclei and glial cell proliferation, suggest significant cellular stress and damage in response to viral infection. These alterations are consistent with findings in other viral encephalitis models (e.g., EV-A71, CV-A6, and E11), where neuronal injury and inflammatory responses contribute to disease severity (22, 25, 26). The presence of perivascular cuffing and localized hemorrhaging indicates a robust inflammatory response that may facilitate viral neuroinvasion and pathogenesis. This aligns with other studies demonstrating how inflammatory mediators can enhance viral spread by disrupting the blood–brain barrier and amplifying neuroinflammatory processes (27–29). The significant lymphocytic infiltration observed further supports the notion that the immune response plays a dual role, potentially aiding in viral clearance while simultaneously exacerbating tissue injury. Immunofluorescence analysis revealed prominent dsRNA signals in the thalamus, meninges, and hippocampus of E6-infected mice, indicating widespread viral dissemination within CNS. This finding is particularly significant as it identifies potential neuroanatomical targets for viral invasion, consistent with previous research on enteroviral neurotropism. However, Li et al. (30), while constructing an E30 infection mouse model, found that viral dsRNA was distributed throughout the entire brain. This comparison emphasizes the potential primary sites of viral activity within the central nervous system in E6 infection.

Cytokine analysis in hFcRn^Tg^ mice infected with E6 reveals a pronounced inflammatory response, characterized by significant elevations in various pro-inflammatory cytokines and chemokines. The marked increase in cytokines such as IL-27, IFN-γ, and TNF-α indicates robust immune activation, which is crucial for combating viral infections (31–33). This heightened immune response is consistent with findings in other viral encephalitis models, where similar cytokine profiles have been associated with severe disease outcomes (34–36). The significant increases in chemokines such as MCP-1, MCP-3, and RANTES suggest enhanced recruitment of immune cells to the site of infection, potentially contributing to the observed tissue damage and inflammation (37–39). Additionally, elevated levels of IL-6 and IL-18 further support the presence of a heightened inflammatory environment, which can lead to neuroinflammation and neuronal damage (40, 41). Overall, these findings underscore the critical role of the inflammatory response in the pathogenesis of E6 infection. Understanding the dynamics of cytokine expression and immune cell recruitment in this model may provide valuable insights into potential therapeutic strategies aimed at modulating the immune response to mitigate tissue damage while promoting viral clearance.

In this study, multi-omics analyses showed that innate immunity, including etinoic acid inducible gene-I (RIG-I) and nuclear factor kappa-light-chain-enhancer of activated B cells (NF-κB), is rapidly activated following E6 infection. Robust immune responses are essential for controlling viral replication and spread within the central nervous system, as demonstrated by many researchers (39, 42, 43). Upon cytosolic entry of viral RNA, retinoic acid-inducible gene I-like receptors (RLRs), such as RIG-I and melanoma differentiation-associated protein 5 (MDA5), recognize RNA and recruit mitochondrial antiviral signaling protein (MAVS), activating downstream pathways (44, 45). This cascade induces type I interferons (IFN-α, IFN-β), key mediators of antiviral defense that engage the Janus kinase (JAK) -STAT pathway to drive antiviral gene expression and suppress viral replication (45). Conversely, the downregulation of metabolic pathways, including tyrosine metabolism and serotonergic synaptic pathways, indicates post-infection metabolic alterations. Accumulating evidence suggests that such metabolic reprogramming in infected cells predominantly benefits viral propagation rather than the host (46). Network analysis identified key proteins, including Myd88, Cxcl10, and Irf3, which are central to the immune response (47–49). Liu et al. (48) demonstrated the significance of MyD88-dependent mechanisms in eliciting maximal pro-inflammatory responses and astrocyte activation (47). These proteins play critical roles in signaling pathways that mediate the host’s antiviral response, reinforcing the interconnectedness of immune signaling during E6 infection. Overall, these changes indicate a coordinated immune-metabolic response to E6 infection. Clarifying these dynamics may inform therapies that modulate host responses to enhance viral clearance while limiting tissue injury.

Interestingly, we observed upregulation of Zbp1, Stat1, Nlrc5, Casp1, and nuclear factor of kappa light polypeptide gene enhancer in B-cells inhibitor alpha (Nfkbia), which showed positive correlations with other elevated inflammatory mediators. These factors, especially ZBP1, were considered key markers regulating PANoptosis (50). Malireddi et al. (50) introduced the concept of PANoptosis, describing an innate immune, lytic, and inflammatory cell death program that coordinately integrates pyroptosis, apoptosis, and necroptosis. Consistent with this framework, Kuriakose et al. (51) found that IAV infection activates ZBP1, which in turn triggers nucleotide-binding oligomerization domain, leucine-rich repeat and pyrin domain-containing protein 3 (NLRP3) inflammasome activation and PANoptosis (52). In 2025, Yang et al. (53) reported that severe acute respiratory syndrome coronavirus 2 (SARS-CoV-2) infection can induce adenosine deaminase acting on RNA 1 (ADAR1) degradation via activation of the cyclic GMP-AMP synthase-stimulator of interferon genes (cGAS-STING) pathway, leading to the accumulation of Z-nucleic acids and subsequent ZBP1-dependent PANoptosis in bystander cells, thereby sustaining inflammation. In addition, Li et al. (54) found that EV-A71 infection induces apoptosis through reactive oxygen species (ROS) and sirtuin 1 (SIRT1) activation; Wang et al. (55) showed that E11 infection activates the NLRP3 inflammasome to drive pyroptosis; and Wang et al. (56) further reported that EV-A71 induced autophagy by inhibiting mammalian target of rapamycin (mTOR) and activating extracellular signal-regulated kinase (ERK) signaling. Taken together with our findings, these studies raise the possibility that EV-induced CNS infections are linked to PANoptosis, although the precise mechanisms remain to be elucidated.

## 5 Conclusion

In summary, the establishment of this hFcRn^Tg^ model represents a significant advancement in understanding E6 pathogenesis and host-pathogen interactions. Our analysis demonstrates that the dynamic interplay between viral infection and host immune responses-characterized by distinct inflammatory profiles and metabolic reprogramming-underscores the complex nature of E6 pathogenesis. Compared to existing immunodeficient models, this model offers distinct advantages by preserving intact immune responses, making it particularly valuable for vaccine development and drug screening. Additionally, our omics data lay a critical foundation for further investigation into the mechanisms of E6 infection. Overall, these findings enhance our understanding of E6-related disease mechanisms and provide a basis for future studies on viral encephalitis and meningitis.

## Data Availability

The original contributions presented in the study are included in the article/Supplementary material, further inquiries can be directed to the corresponding author.
